# Docetaxel Rechallenge After Chemohormonal Therapy in High-Volume Metastatic Castration-Sensitive Prostate Cancer

**DOI:** 10.7759/cureus.91143

**Published:** 2025-08-27

**Authors:** Kenichi Hata, Yuki Takiguchi, Yuya Iwamoto, Taisuke Yamazaki, Takahiro Kimura

**Affiliations:** 1 Department of Urology, Shonan Keiiku Hospital, Fujisawa, JPN; 2 Department of Urology, Atsugi City Hospital, Atsugi, JPN; 3 Department of Urology, The Jikei University School of Medicine, Tokyo, JPN

**Keywords:** chemohormonal therapy, docetaxel, metastatic castration-resistant prostate cancer, metastatic castration-sensitive prostate cancer, rechallenge

## Abstract

Docetaxel has been widely used in the treatment of both metastatic castration-resistant and castration-sensitive prostate cancers. However, limited evidence exists regarding the efficacy of docetaxel rechallenge (DR) in patients with metastatic castration-resistant prostate cancer (mCRPC) following prior chemohormonal therapy for high-volume metastatic castration-sensitive prostate cancer (mCSPC). This study aimed to evaluate clinical outcomes in such patients. Among 32 patients with high-volume mCSPC treated with chemohormonal therapy between 2020 and 2022, five underwent DR after progression to mCRPC. A retrospective review was performed to assess patient characteristics, changes in prostate-specific antigen (PSA) levels, and treatment-related adverse events. The median follow-up period was 26 months, and the median age at DR initiation was 74 years. The median time to progression to mCRPC was 27 months (range: 7-41 months). DR was administered as either first- or second-line therapy for mCRPC. Treatment was discontinued in two patients due to disease progression. A ≥50% decline in PSA levels was observed in two patients who received docetaxel as the first-line mCRPC therapy (40%). Grade 3 fatigue occurred in two patients, with low-grade alopecia being the most common adverse event. DR may provide limited clinical benefits in selected patients with mCRPC following prior chemohormonal therapy, particularly when treatment options are constrained. However, due to the small size and variability in response, the therapeutic role of the modality remains uncertain. Well-designed prospective studies with larger cohorts are needed to better define the clinical value of DR and establish evidence-based criteria for patient selection.

## Introduction

Three consecutive phase III randomized, placebo-controlled trials demonstrated that the combination of docetaxel (DTX) and androgen deprivation therapy (ADT) significantly improves overall survival in patients with metastatic castration-sensitive prostate cancer (mCSPC) compared with ADT monotherapy [1-3]. As a result, chemohormonal therapy has been established as the standard of care for mCSPC over the past decade. However, this therapeutic approach was removed from the National Comprehensive Cancer Network Clinical Practice Guidelines following the approval of triplet regimens, which consist of ADT combined with DTX and either abiraterone acetate or darolutamide, based on the findings of the PEACE-1 and ARASENS trials [4,5]. With the advent of these triplet regimens, the use of DTX in mCSPC is expected to increase. Among patients who experienced disease progression, DTX rechallenge (DR) was employed in 21% to 30% of cases as part of sequential therapy [4,5].

A retrospective analysis of the GETUG-AFU 15 trial found that DR administered following progression to castration resistance was effective in only a limited number of patients previously treated with chemohormonal therapy for mCSPC [6]. By contrast, Mahler et al. analyzed data from a Canadian cohort of 55 patients with mCSPC who received chemohormonal therapy followed by DR during the castration-resistant phase. Their findings indicated that more than a quarter of these patients experienced a prostate-specific antigen (PSA) decline of ≥50% [7]. The median progression-free survival during DR was 4.1 months across the cohort, with prolonged disease control observed in patients with a Gleason score of ≥8. Despite prior observations, the evidence base remains fragmented and primarily descriptive, lacking a hypothesis-driven framework to guide patient selection and therapeutic decision-making. Accordingly, this study aimed to assess whether DR confers meaningful clinical benefit in patients with metastatic castration-resistant prostate cancer (mCRPC) previously treated with chemohormonal therapy for mCSPC and to identify clinical patterns that may inform future prospective investigations.

## Case presentation

Materials and methods

Among 32 patients with high-volume mCSPC who received chemohormonal therapy with ADT and DTX at Atsugi City Hospital between 2015 and 2022, 20 subsequently progressed to mCRPC. A retrospective review was conducted on five patients with mCRPC who underwent DR therapy. Patient selection for DR was determined not only by clinical criteria but also by individual preferences and socioeconomic factors. These real-world considerations may have contributed to the limited number of patients included in the rechallenge cohorts. Demographic and clinical data were manually extracted from institutional medical records, including age, PSA level at diagnosis, Gleason score, presence of visceral and lymph node metastases, extent of disease (EOD) score, number of prior DTX cycles, time to progression to CRPC, sequence of DR, PSA level at DR initiation, time to progression, follow-up duration, and oncological outcomes. Patients were generally followed every one to three months via clinical history review, physical examination, routine blood testing, and serum chemistry analysis. Thoracoabdominopelvic computed tomography (CT) and/or bone scintigraphy were performed every 6-12 months or earlier if clinically indicated.

Case presentation

Patient 1

**Figure 1:**
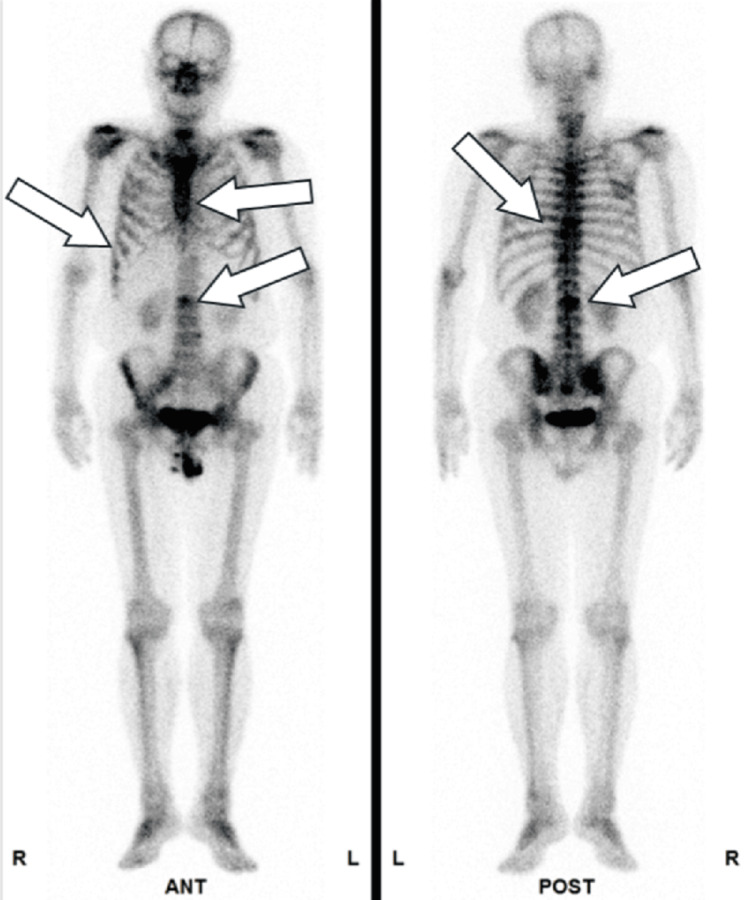
Metastatic site in patient 1 Bone scintigraphy revealed multiple metastatic lesions in the ribs, thoracic vertebrae, and lumbar vertebrae, as indicated by arrows.

**Figure 2:**
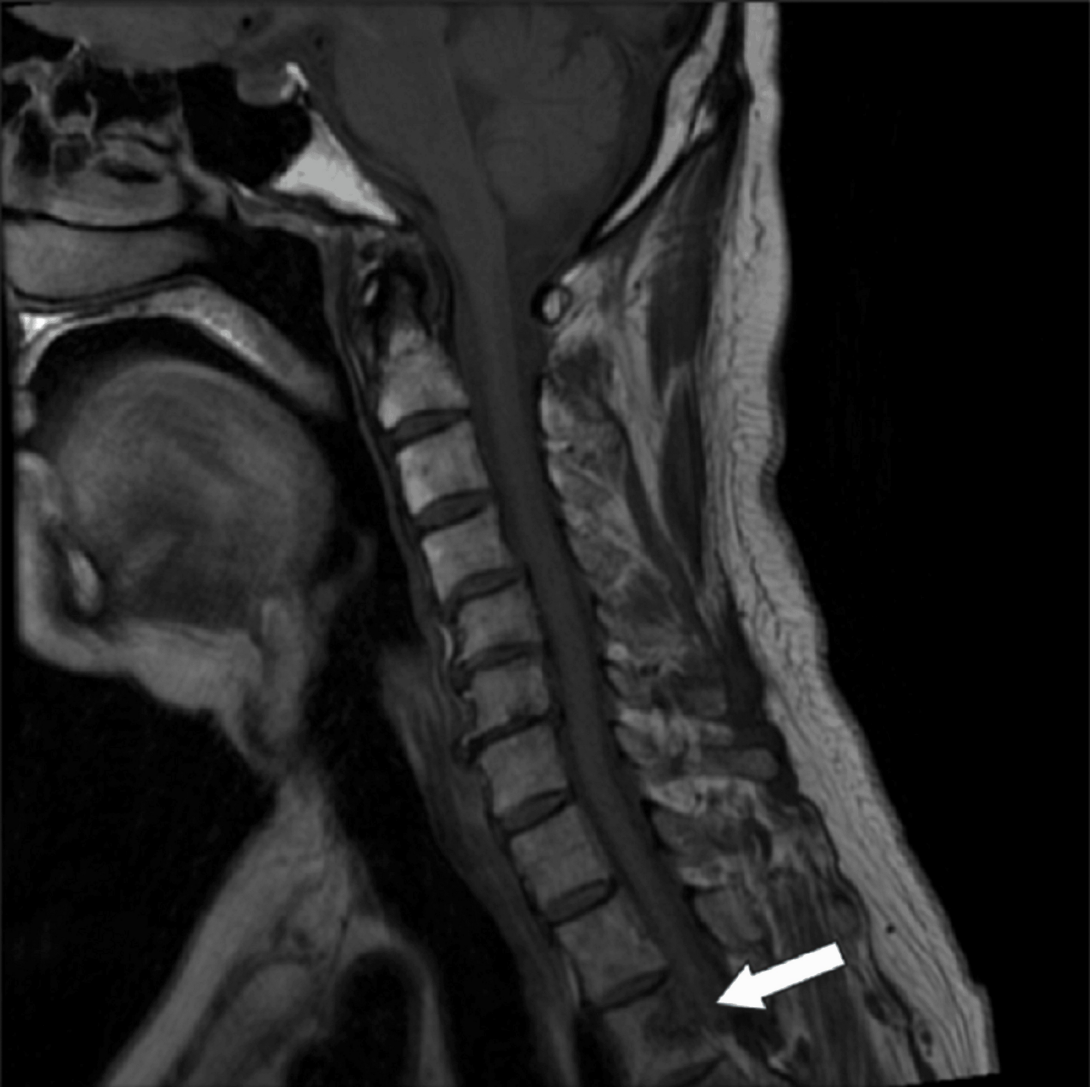
Metastatic sites in patient 1 Magnetic resonance imaging demonstrated thoracic vertebral metastasis, as indicated by arrows.

**Figure 3:**
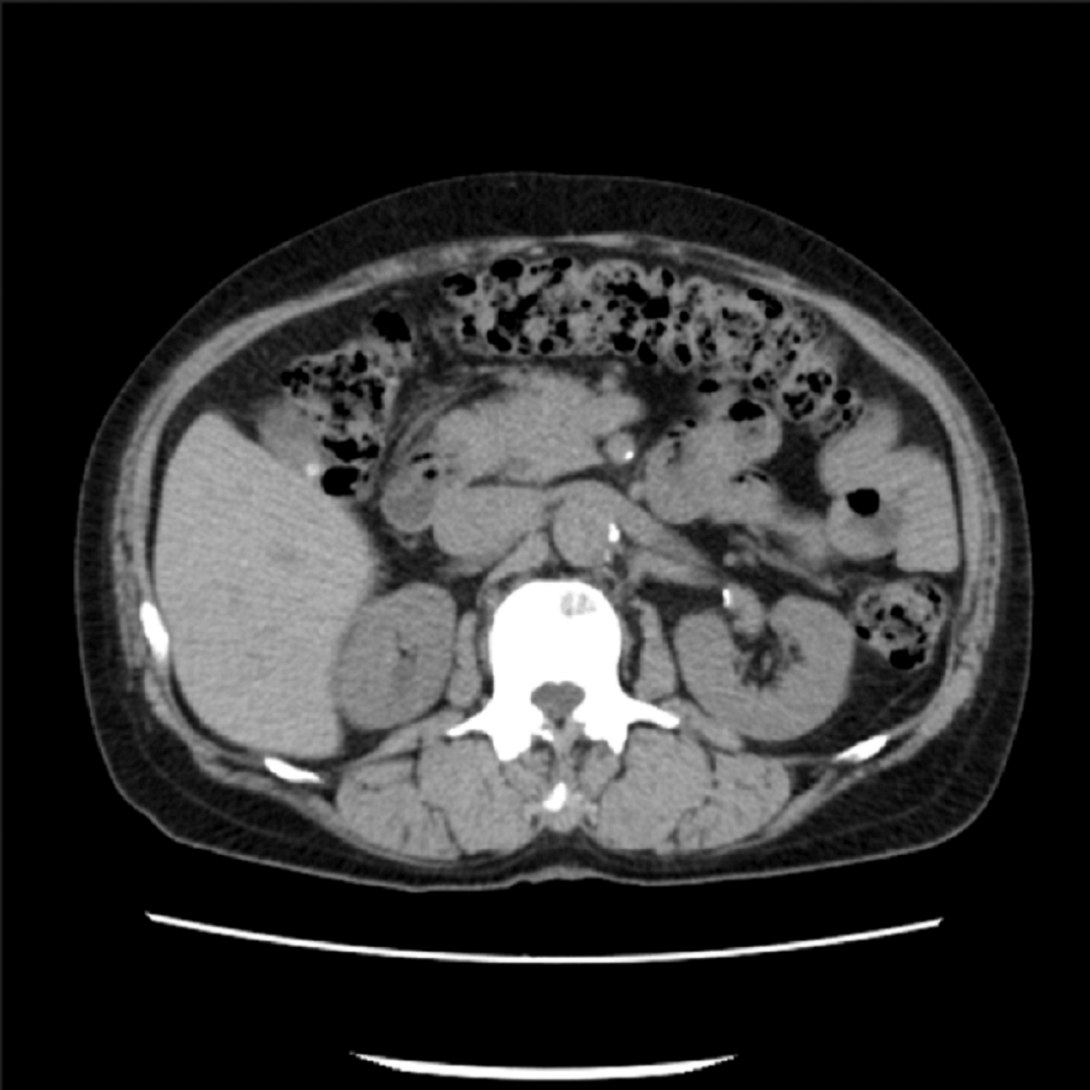
Metastatic sites in patient 1 Computed tomography imaging revealed no apparent visceral metastases.

A 69-year-old man was referred to Atsugi City Hospital with a three-month history of nocturia. His medical history included hypertension and diabetes mellitus, with no known family history of malignancy. Laboratory investigations revealed a PSA level of 250 ng/mL. Transrectal ultrasound-guided prostate biopsy (TR-PB) confirmed adenocarcinoma with a Gleason score of 8. Subsequent radiographic assessment led to a diagnosis of clinical stage T4N1M1b (stage D2) prostate cancer. The patient received initial chemohormonal therapy consisting of DTX at 75 mg/m2 every four weeks for six cycles, in combination with ADT using a luteinizing hormone-releasing hormone (LH-RH) agonist and bicalutamide (80 mg/day). Twenty-one months after completion of DTX chemotherapy, the patient progressed to mCRPC, indicated by increased PSA and imaging-confirmed bone metastases. First-line treatment for mCRPC included abiraterone acetate (ABI) at 1000 mg/day with prednisone 10 mg/day. Although there was an initial decline in PSA levels, they subsequently increased to 12.01 ng/mL, accompanied by radiologic evidence of disease progression (Figures 1-3). Second-line treatment with DR was initiated; however, after three cycles, PSA levels failed to decline, and progression of bone metastases persisted. Consequently, DR was discontinued, and the patient was transitioned to cabazitaxel (CBZ) chemotherapy.

Patient 2

**Figure 4:**
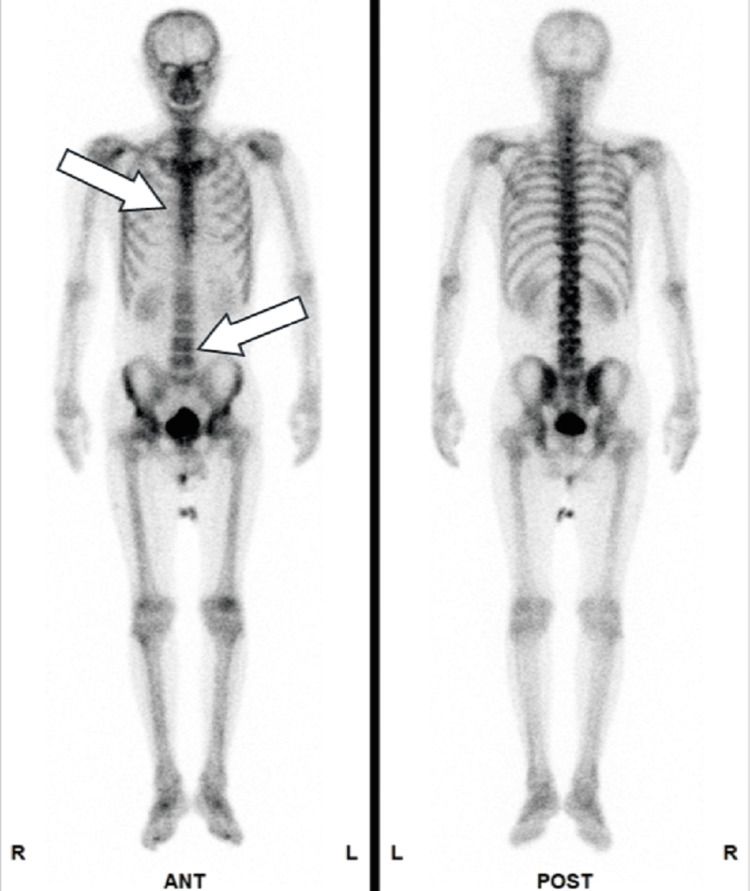
Metastatic sites in patient 2 Bone scintigraphy revealed multiple metastatic lesions in the thoracic and lumbar vertebrae, as indicated by arrows.

**Figure 5:**
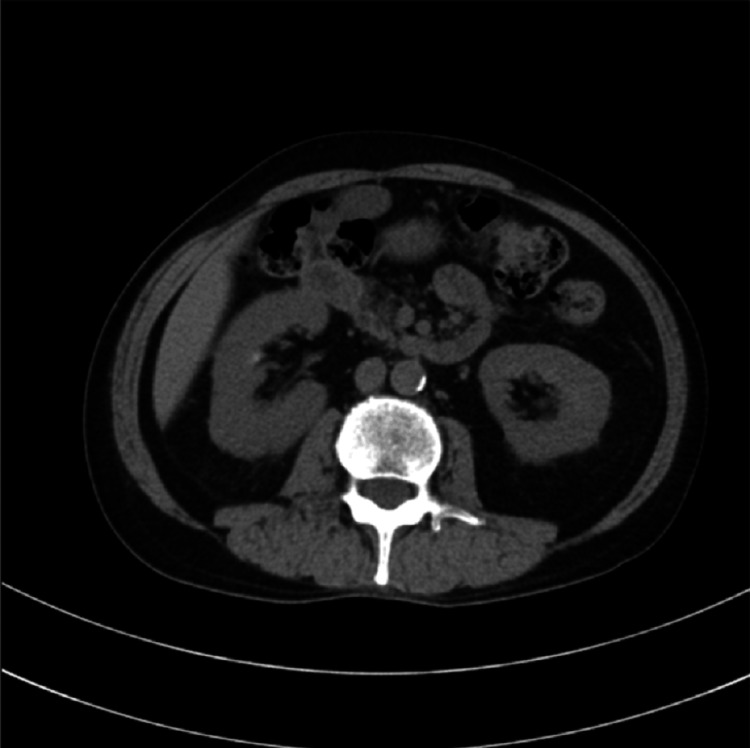
Metastatic sites in patient 2 Computed tomography imaging revealed no apparent visceral metastases.

A 53-year-old man presented to Atsugi City Hospital with a chief complaint of frequent urination. His medical history included diabetes mellitus with poor glycemic control and no significant family history of malignancy. Laboratory testing revealed an elevated PSA level of 17.89 ng/mL, and a TR-PB confirmed adenocarcinoma with a Gleason score of 10. Imaging demonstrated right obturator lymph node involvement and high-volume bone metastases, consistent with a diagnosis of clinical stage T3bN1M1b (stage D2) prostate cancer. The patient was initiated on short-term bicalutamide therapy (80 mg/day), followed by surgical castration. Two weeks later, he received six cycles of DTX chemotherapy, administered every four weeks. He remained recurrence-free for 35 months. However, a subsequent increase in PSA to 4.45 ng/mL and new-onset bone pain indicated disease progression, with imaging confirming progression of bone metastases (Figures 4, 5). Ten cycles of DR therapy were then administered. Although the patient achieved an 18-month recurrence-free interval after DR therapy, the disease subsequently recurred. Treatment was transitioned to first-generation antiandrogen therapy; however, the patient died 34 months after the initiation of DR.

Patient 3

**Figure 6:**
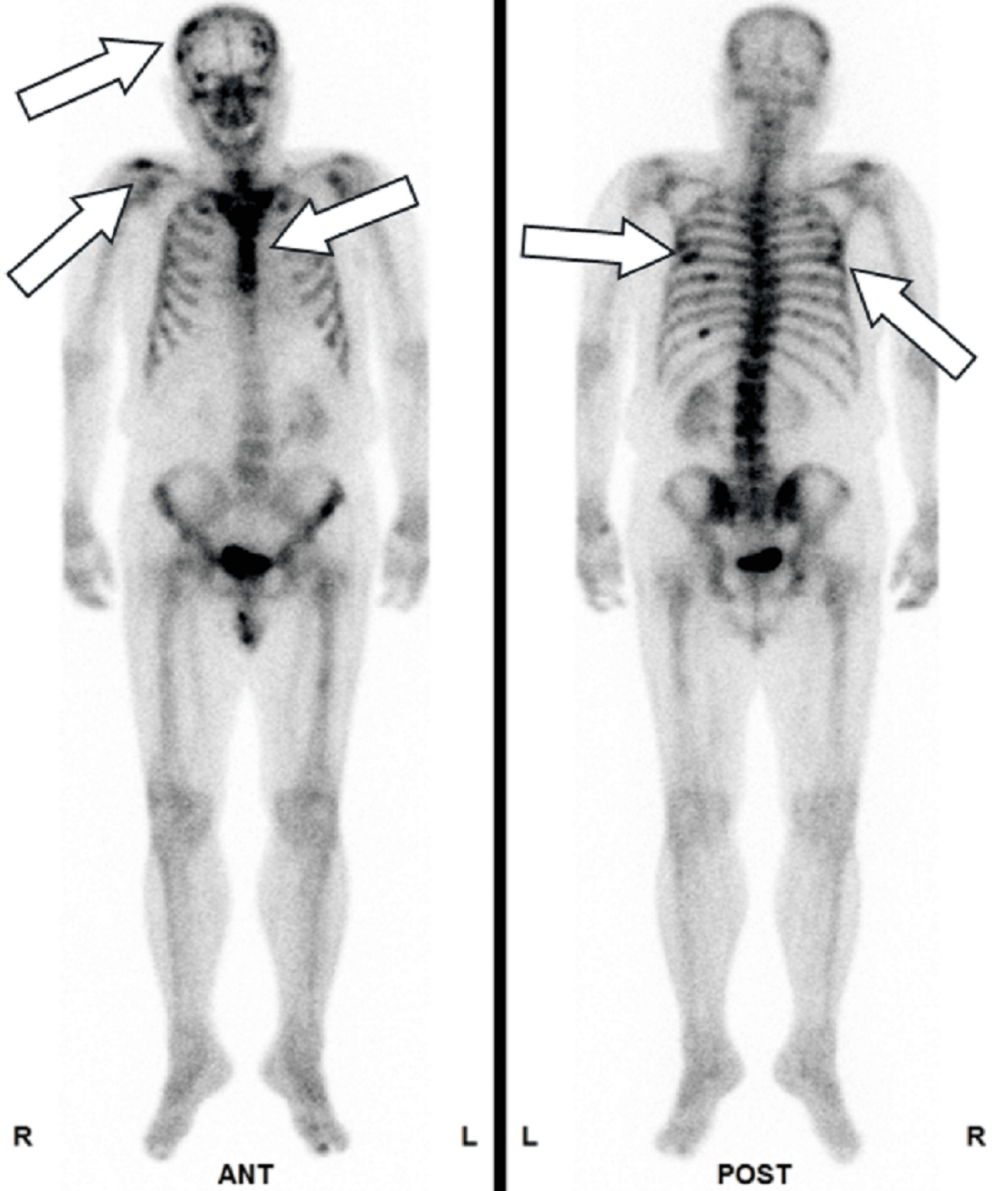
Metastatic sites in patient 3 Bone scintigraphy revealed multiple metastatic lesions in the cranial bones, clavicle, ribs, and lumbar vertebrae, as indicated by arrows.

**Figure 7:**
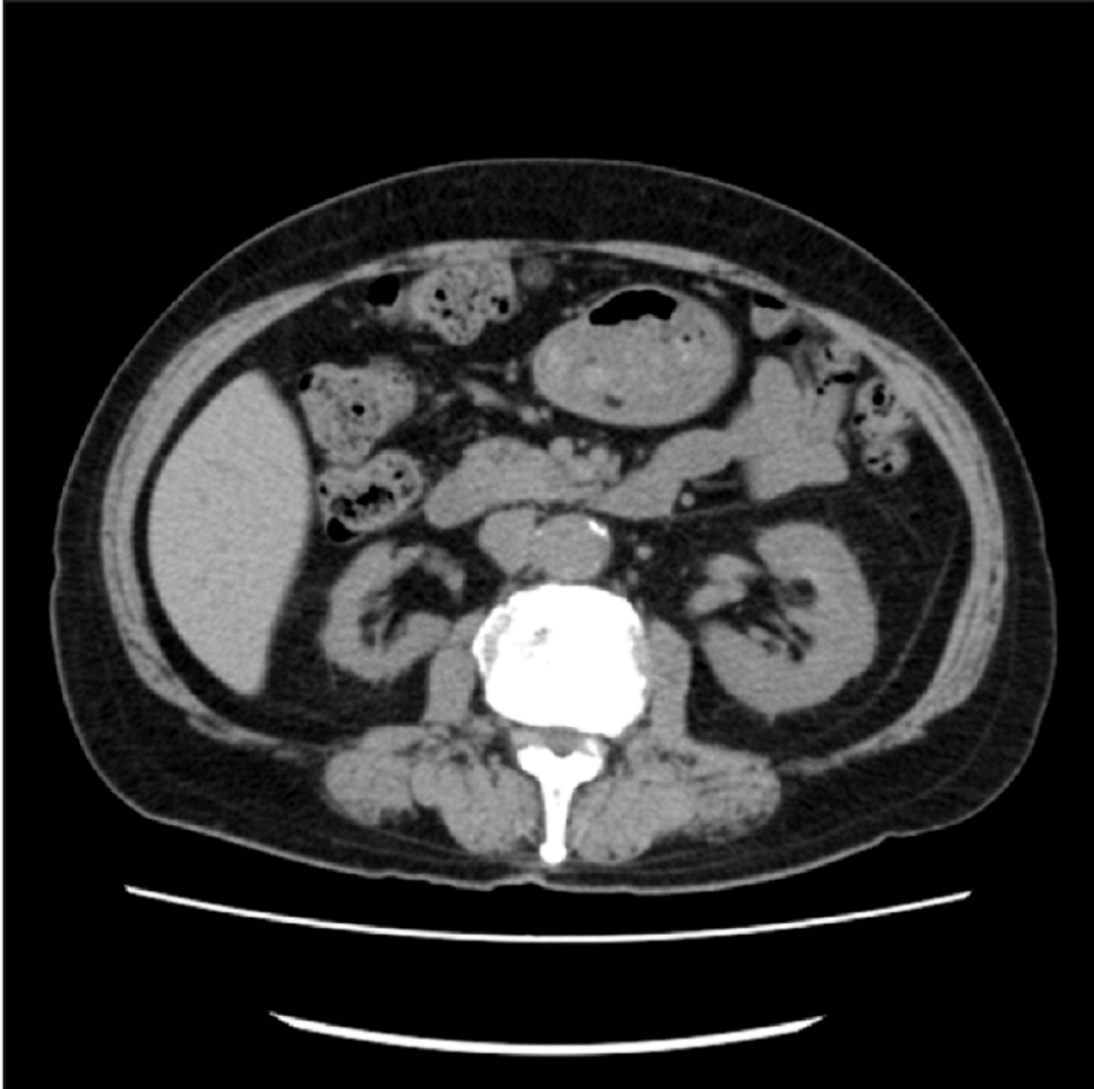
Metastatic sites in patient 3 Computed tomography imaging revealed no apparent visceral metastases.

A 73-year-old man presented to Atsugi City Hospital with a chief complaint of right flank pain. His medical history included lacunar infarction and bronchial asthma, with no significant family medical history of malignancy. CT revealed multiple lymph node metastases and widespread bone metastases, with a PSA level of 3817 ng/mL. TR-PB confirmed adenocarcinoma with a Gleason score of 10. Further evaluation using bone scintigraphy demonstrated high-volume bone metastases, consistent with a diagnosis of clinical stage T4N1M1b (stage D2) prostate cancer. Chemohormonal therapy was initiated, consisting of ADT using an LH-RH analog in combination with DTX chemotherapy at 75 mg/m2 every three weeks for six cycles. Following treatment, opioid analgesics were successfully discontinued, indicating adequate control of cancer-related pain. However, 14 months after initiating therapy, progression to mCRPC was observed. First-line treatment with ABI (1000 mg/day) and prednisone (10 mg/day) initially led to a reduction in PSA; however, PSA levels subsequently increased to 157 ng/mL, and imaging confirmed disease progression at 15 months, necessitating a switch to DR therapy (Figures 6, 7). After three cycles of DR, the PSA level escalated to 310 ng/mL, accompanied by a recurrence of worsening cancer-related pain. Consequently, the patient was transitioned to CBZ chemotherapy as third-line treatment for mCRPC.

Patient 4

**Figure 8:**
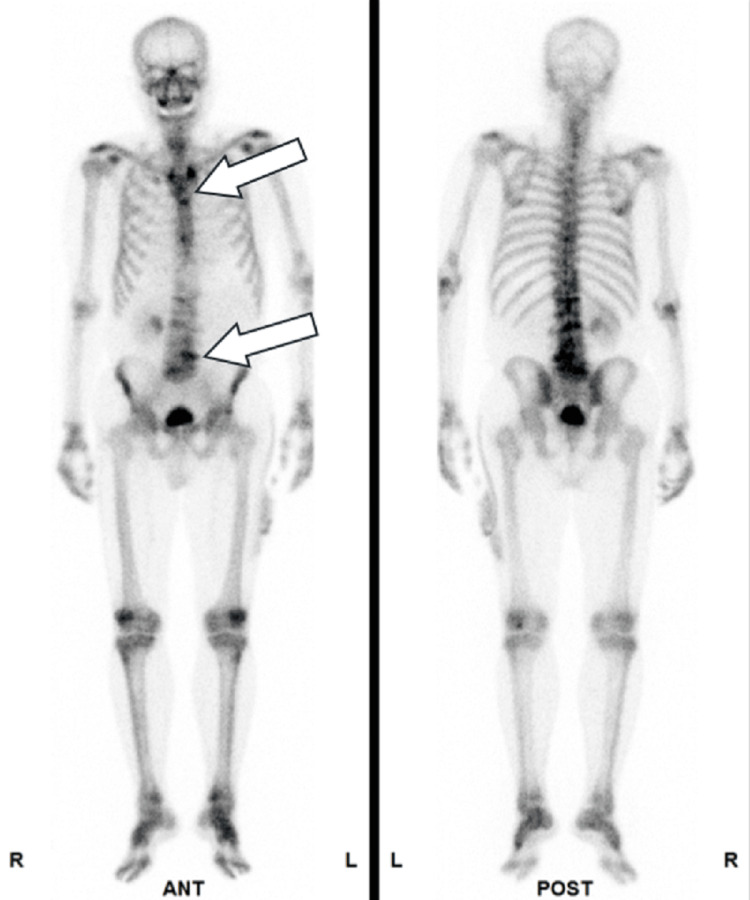
Metastatic sites in patient 4 Bone scintigraphy revealed multiple metastatic lesions in the thoracic and lumbar vertebrae, as indicated by arrows.

**Figure 9:**
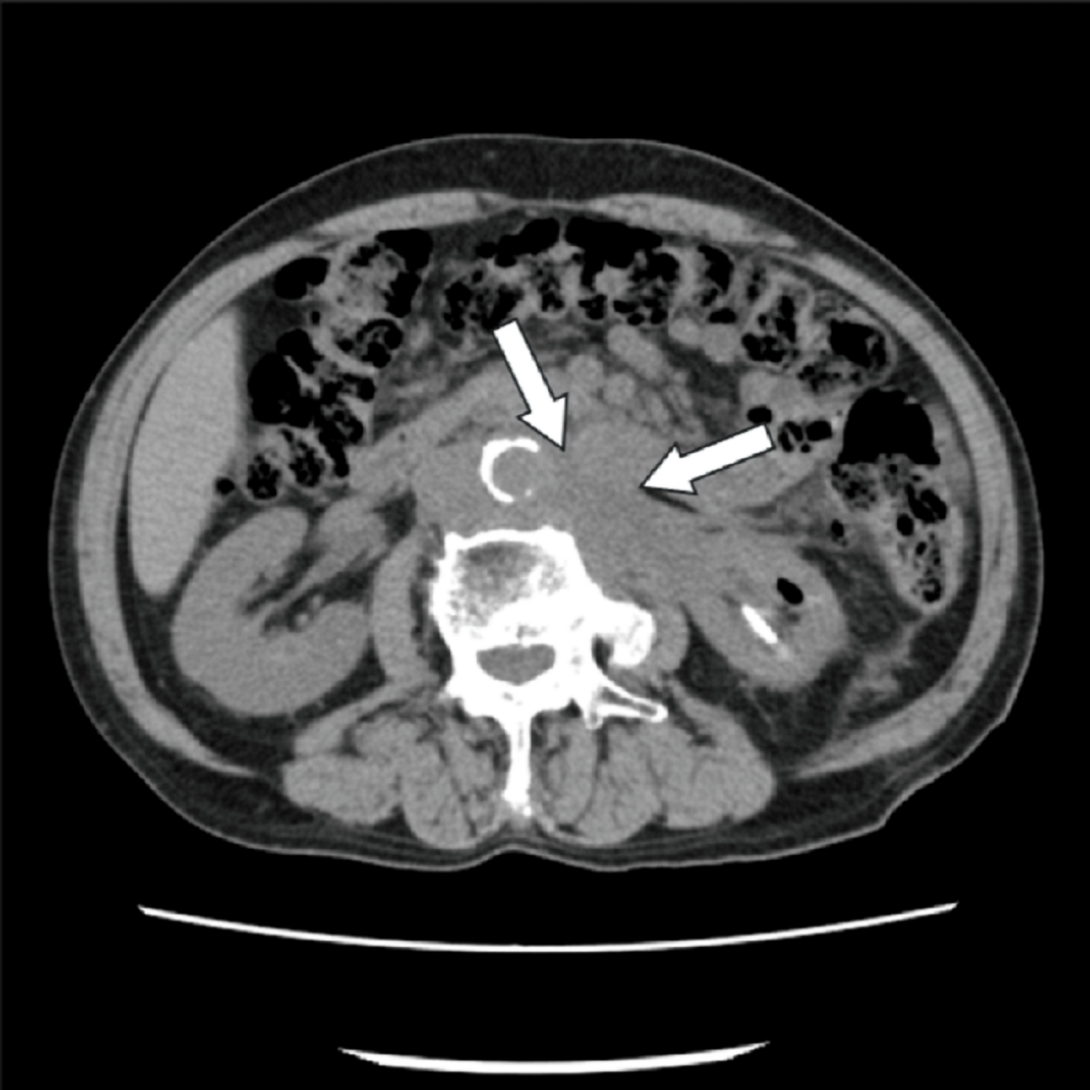
Metastatic sites in patient 4 Computed tomography imaging demonstrated para-aortic lymph node metastases, as indicated by arrows. A left percutaneous nephrostomy was subsequently performed.

A 67-year-old man presented to Atsugi City Hospital with complaints of left flank pain. His medical and family history revealed no significant findings. CT imaging suggested left hydronephrosis caused by metastases in the para-aortic and bilateral obturator lymph nodes, along with multiple bone metastases. To differentiate potential malignancies, his PSA level was measured and found to be markedly elevated at 294 ng/mL. Concurrently, a percutaneous left nephrostomy was performed. TR-PB revealed adenocarcinoma with a Gleason score of 9. Additional bone scintigraphy confirmed high-volume bone metastases, consistent with a diagnosis of clinical T4N1M1b, stage D2 prostate cancer. Two weeks post-surgical castration, six cycles of DTX chemotherapy, administered every three weeks. After seven months, PSA levels increased to 46.75 ng/mL, with imaging confirming progression of lymph node and bone metastases. DR therapy was initiated (Figures 8, 9). Following seven cycles of DR, the patient developed grade 3 fatigue, as assessed using the Common Terminology Criteria for Adverse Events (CTCAE) version 5, necessitating discontinuation of treatment [8]. Subsequently, second-line therapy with ABI was initiated.

Patient 5

**Figure 10:**
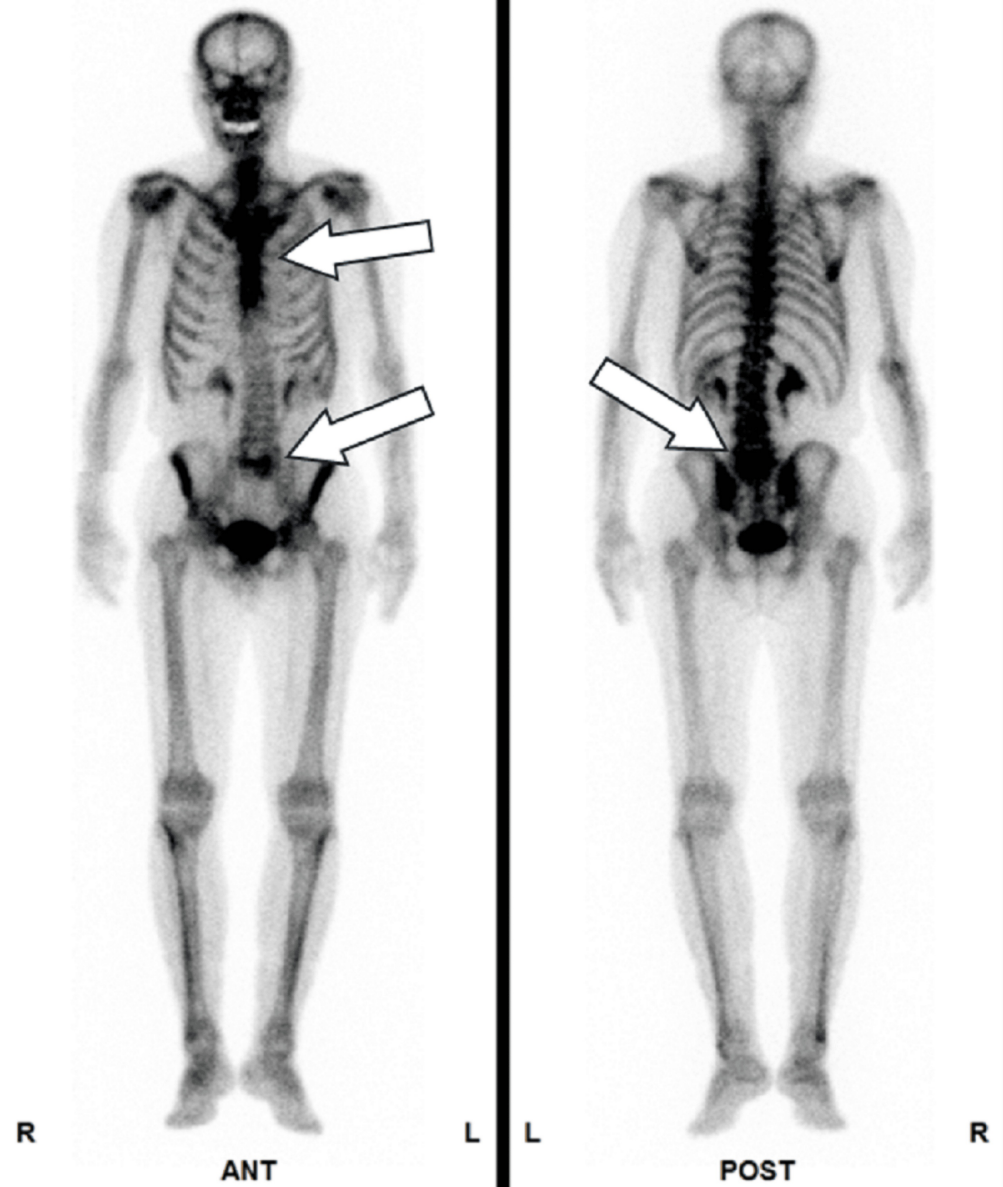
Metastatic sites in patient 5 Bone scintigraphy revealed multiple metastatic lesions in the pelvic bone, thoracic vertebrae, and lumbar vertebrae, as indicated by arrows.

**Figure 11:**
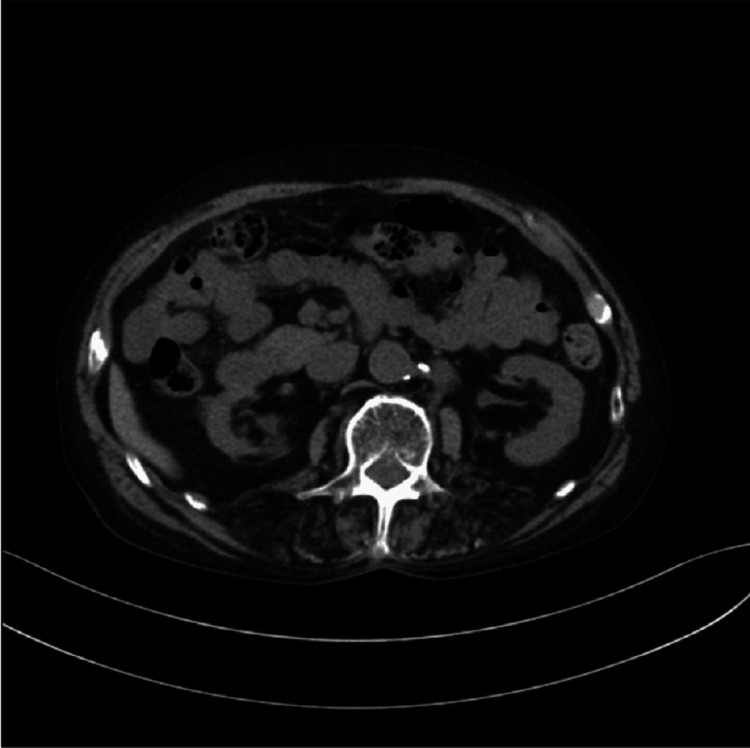
Metastatic sites in patient 5 Computed tomography imaging revealed no apparent visceral metastases.

A 70-year-old man presented to Atsugi City Hospital with nocturia as the chief complaint. No relevant past medical or family history was noted. CT imaging revealed bilateral obturator lymphadenopathy and bilateral hydronephrosis secondary to a prostate tumor infiltrating the bladder. The patient had a PSA level of 653 ng/mL, and TR-PB confirmed adenocarcinoma with a Gleason score of 8. Simultaneously, bilateral percutaneous nephrostomy was performed. Bone scintigraphy revealed high-volume metastases to the pubis and ischium. Based on these findings, the patient was diagnosed with clinical stage T4N1M1b (stage D2) prostate cancer. Initial treatment comprised ADT using an LH-RH antagonist and bicalutamide, in combination with DTX chemotherapy (75 mg/m2 every three weeks for six cycles). The prostate tumor responded significantly, resulting in the resolution of bilateral hydronephrosis and enabling the removal of the nephrostomy catheters. At 33 months post-treatment, disease progression to mCRPC was confirmed. ABI (1000 mg/day) was introduced as first-line therapy. Although the patient maintained a clinical response for 27 months, radiological progression was subsequently observed, prompting a transition to DR (Figures 10, 11). After 10 cycles of DR, the best supportive care was subsequently initiated.

Results

Patient demographics and clinical characteristics are summarized in Table 1. All patients presented with high-volume metastatic disease, as defined by the Chemohormonal Therapy Versus Androgen Ablation Randomized Trial for Extensive Disease in Prostate Cancer criteria, and had a high Gleason score of ≥8 at diagnosis [2]. All patients completed six cycles of DTX treatment for mCSPC and achieved a PSA decline of ≥90% from baseline following chemohormonal therapy. The time to progression to CRPC ranged from 7 to 41 months (median: 27 months). The median follow-up duration was 26 months (Table 1). Primary prophylaxis with granulocyte colony-stimulating factor and oral corticosteroids was mandatory during DR administration.

**Table 1:** Baseline demographics and clinical characteristics of the study patients PSA: prostate-specific antigen; EOD: extent of disease; DTX: docetaxel; DR: docetaxel rechallenge; CRPC: castration-resistant prostate cancer.

Case	Age	PSA at diagnosis (ng/mL)	Gleason score	Visceral metastasis	Lymph node metastasis	EOD score	Cycles of prior DTX	Time to CRPC (months)	DR sequence	PSA at DR (ng/mL)	Cycles of DR	Time to progression (months)	Follow-up (months)	Outcome
															1	74	250	8	None	Present	3	6	27	2^nd^ line	12.01	3	3	26	Death
2	57	17.89	10	None	Present	1	6	41	1^st^ line	4.45	10	18	34	Death
3	75	3817	10	None	Present	4	6	14	2^nd^ line	157	3	2	25	Death
4	69	294	9	None	Present	3	6	7	1^st^ line	46.75	7	8	49	Alive
5	76	653.8	8	None	Present	1	6	33	2^nd^ line	4.61	10	11	16	Alive

**Figure 12:**
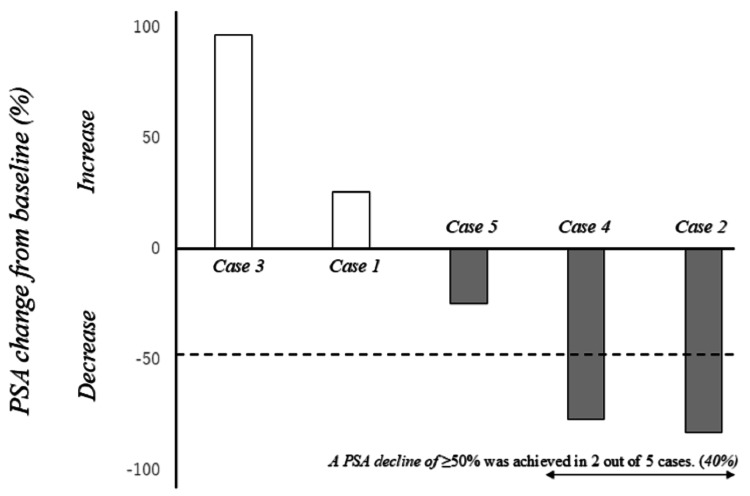
PSA decline after docetaxel rechallenge was used as a first- or second-line therapy in patients with metastatic castration-resistant prostate cancer. As indicated by the arrows, a PSA decline of ≥50% was observed in two of the five patients (40%). PSA: prostate-specific antigen

All patients received DR as either first- or second-line salvage therapy for mCRPC. At the time of mCRPC diagnosis, all patients exhibited multiple bone metastases without visceral involvement. However, in patient 4, bulky para-aortic lymph node metastases were identified, and percutaneous nephrostomy was performed due to left-sided hydronephrosis caused by these lesions (Figures 8, 9). Treatment with DR was discontinued in two patients after three cycles due to disease progression without any decline in PSA (Table 1). A PSA decline of ≥50% was achieved in two of the five patients (40%) (Figure 12). These two patients were relatively younger compared with the others and had received DR as first-line therapy for mCRPC. Furthermore, the time to CRPC progression differed considerably between these two patients (7 months vs. 41 months). During the observation period, three patients died due to disease progression, and one patient was lost to follow-up after receiving the best supportive care at another hospital. All patients who underwent DR experienced grade 1-2 adverse events. However, grade 3 fatigue was reported in two patients, one of whom discontinued treatment after seven cycles. The most frequently reported adverse event was alopecia, which occurred in four out of five patients (80%) (Table 2).

**Table 2:** Adverse events in five patients who received docetaxel rechallenge for metastatic castration-resistant prostate cancer Proportion of adverse events linked to docetaxel rechallenge, categorized using the Common Terminology Criteria for Adverse Events (CTCAE) version 5.

Number of patients (%)	Adverse events for docetaxel rechallenge (n=5)	
	Grade 1-2	Grade 3-5
Fatigue	0 (0)	2 (40)
Diarrhea	1 (20)	0
Nausea, vomiting, or both	1 (20)	0
Changes in taste	3 (60)	0
Stomatitis	1 (20)	0
Constipation	1 (20)	0
Peripheral edema	2 (40)	0
Nail changes	2 (40)	0
Alopecia	4 (80)	0
Arthralgia	3 (60)	0
Peripheral neuropathy	3 (60)	0
Anemia	3 (60)	0

## Discussion

DTX was the first systemic therapy demonstrated to confer a survival benefit in patients with mCSPC and mCRPC [1-3,9]. Several retrospective studies have shown that the proportion of patients with mCRPC who received DR and experienced a PSA decline of ≥50% following first-line DTX treatment ranged from 22% to 66% [10-13]. However, no prospective phase III studies have investigated the efficacy of DR in patients with mCRPC following disease progression. To date, Di Lorenzo et al. have conducted the only prospective phase II study investigating 45 patients with mCRPC who received DR after achieving a complete biochemical response to first-line DTX chemotherapy [14]. Among them, 11 patients (24.4%) achieved a PSA decline of ≥50%. Objective responses were observed in 4 of 16 patients (25%) with measurable disease. The median progression-free survival was five months, whereas the median overall survival was 13 months. The most frequently reported grade 3 toxicity was neutropenia, which occurred in 17.8% of the patients. Caffo et al. reported that the slope of interval log PSA, duration of the preceding treatment-free interval, and response to the previous DR cycle were predictive of subsequent DR response. Based on these findings, DR was concluded to preserve antitumor activity and to be well-tolerated in a selected patient population [15]. Notably, a retrospective analysis by Hung et al. compared patients who received either first-line DTX or DR after treatment with androgen receptor-signaling inhibitors with those who did not receive DR. The overall survival was significantly improved in the DR group compared with the non-DR group (50.11 months vs. 26.36 months, respectively, p = 0.044) [16].

To date, no studies have reported on the efficacy of DR following triplet therapy, although several studies have evaluated DR after chemohormonal therapy [6,7,17,18]. At the Advanced Prostate Cancer Consensus Conference, no consensus was reached regarding the use of DR in most patients with mCSPC who progressed to mCRPC within 12 months of initial DTX treatment. However, consensus was achieved that DR may be considered appropriate for patients whose progression to mCRPC occurs beyond 36 months [19]. The first study to investigate the efficacy of DR following disease progression after prior chemohormonal therapy was conducted by Lavaud et al., presented as a subgroup analysis of the GETUG-AFU 15 phase Ⅲ trial [6]. Among patients with mCRPC who received DR as first- or second-line therapy, a ≥50% PSA decline was observed less frequently in those previously treated with chemohormonal therapy for mCSPC compared with those who received ADT alone (14% vs. 45%; p = 0.07). DR following progression to mCRPC after chemohormonal therapy for mCSPC was concluded to be effective in only a limited subset of patients. However, the study did not identify specific patient characteristics associated with greater benefits. To further assess the efficacy of DR, a retrospective study was conducted by Mahler et al., in which 54 evaluable patients were analyzed. Among them, 33 patients (61.1%) demonstrated a PSA response, with 15 patients (27.8%) achieving a ≥50% decline in PSA levels. A Gleason score of ≥8 was predictive of prolonged progression-free survival during DR (hazard ratio, 0.32; 95% confidence interval, 0.12-0.81; p = 0.02), although it did not predict the PSA response [7]. In this cohort, the rate of PSA decline of ≥50% was more favorable compared to those reported in previous prospective and retrospective studies. Notably, a favorable PSA response was also observed even in patients with a shorter duration to CRPC, which contrasts with the recommendation of the Advanced Prostate Cancer Consensus Conference that DR should be reserved for those whose progression to mCRPC occurs after 36 months. These findings suggest that the interval to CRPC may not be a reliable predictor of DR efficacy.

The development of DTX resistance in prostate cancer has been attributed to multiple signaling pathways, including alterations in non-coding RNAs, transcription factors, and metabolic regulators. Long non-coding RNAs, such as PCAT1 and NEAT1, modulate the expression of miRNAs (e.g., miR-25-3p, miR-34a-5p, and miR-204-5p), thereby influencing downstream targets, such as SLC4A11 and ACSL4, ultimately contributing to chemoresistance. Circular RNA circ-Foxo3 enhances resistance by regulating Foxo3 expression. Conversely, tumor suppressor pathways mediated by miR-323/p73 and miR-129-5p/CAMK2N1 have been associated with increased sensitivity to DTX. Resistance has also been promoted through metabolic reprogramming via the miR-122/PKM2 axis, which facilitates enhanced glycolysis. Additionally, autophagic processes regulated by the LKB1/AMPK, PrLZ, and KLF5/BECLIN-1 pathways have been implicated in modulating both sensitivity and resistance to DTX. Autophagic activity is modulated by the interplay among JNK, BCL-2, and BECLIN-1. These findings highlight the complexity of the regulatory network underlying DTX resistance and suggest potential therapeutic targets for overcoming chemoresistance in prostate cancer. When therapeutics are introduced to reverse DTX resistance or enhance the efficacy of DTX chemotherapy in patients, safety and biocompatibility should be considered [20]. Once such therapeutic strategies have been established, the efficacy of DR can be more robustly validated.

This study has some limitations. First, this was a case series, which inherently lacks a control group and randomization, limiting the ability to establish causality. Second, this was a retrospective, single-center study in which drug selection was primarily based on the discretion of the attending physician.

## Conclusions

In this real-world retrospective case series, a PSA decline of ≥50% was observed in two of five patients (40%). The introduction of DR as a first-line treatment in the castration-resistant setting following prior therapy suggests potential PSA responses in a subset of patients. Although this strategy may be considered in carefully selected cases, its clinical utility remains uncertain due to the limited efficacy signals and small sample size. To clarify the therapeutic relevance of DR and identify predictive biomarkers, well-designed prospective studies with substantially larger patient cohorts are urgently required.
